# *Helicobacter pylori* colonization and obesity – a Mendelian randomization study

**DOI:** 10.1038/s41598-017-14106-4

**Published:** 2017-10-31

**Authors:** Wouter J. den Hollander, Linda Broer, Claudia Schurmann, David Meyre, Caroline M. den Hoed, Julia Mayerle, Albert Hofman, Georg Homuth, André G. Uitterlinden, Markus M. Lerch, Ernst J. Kuipers

**Affiliations:** 1 Department of Gastroenterology and Hepatology, Erasmus MC University Medical Centre, Rotterdam, The Netherlands; 2 Internal Medicine, Erasmus MC University Medical Centre, Rotterdam, The Netherlands; 3 The Charles Bronfman Institute for Personalized Medicine, Icahn School of Medicine at Mount Sinai, New York, NY USA; 4 The Genetics of Obesity and Related Metabolic Traits Program, Icahn School of Medicine at Mount Sinai, New York, NY USA; 5 Department of Health Research Methods, Evidence, and Impact, McMaster University, Hamilton, Canada; 6 Department of Pathology and Molecular Medicine, McMaster University, Hamilton, Canada; 7 Department of Medicine A, University Medicine Greifswald, Greifswald, Germany; 8 Epidemiology, Erasmus MC University Medical Centre, Rotterdam, The Netherlands; 9 Interfaculty Institute for Genetics and Functional Genomics, University Medicine Greifswald, Greifswald, Germany; 10 Department of Epidemiology, Harvard T.H. Chan School of Public Health, Boston, MA USA

## Abstract

Obesity is associated with substantial morbidity, costs, and decreased life expectancy, and continues to rise worldwide. While etiological understanding is needed for prevention, epidemiological studies indicated that colonization with *Helicobacter pylori (H*. *pylori)* may affect body mass index (BMI), but with inconsistent results. Here, we examine the relationship between *H*. *pylori* colonization and BMI/obesity. Cross-sectional analyses were performed in two independent population-based cohorts of elderly from the Netherlands and Germany (n = 13,044). Genetic risk scores were conducted based on genetic loci associated with either *H*. *pylori* colonization or BMI/obesity. We performed a bi-directional Mendelian randomization. Meta-analysis of cross-sectional data revealed no association between anti-*H*. *pylori* IgG titer and BMI, nor of *H*. *pylori* positivity and BMI. Anti-*H*. *pylori* IgG titer was negatively associated with obesity (OR 0.99972; 95% CI 0.99946-0.99997, p = 0.03) and with obesity classes (Beta −6.91 •10^−5^; 95% CI −1.38•10^−4^, −5.49•10^−7^, p = 0.048), but the magnitude of these effects was limited. Mendelian randomization showed no causal relation between *H*. *pylori* genetic risk score and BMI/obesity, nor between BMI or obesity genetic risk scores and *H*. *pylori* positivity. This study provides no evidence for a clinically relevant association between *H*. *pylori* and BMI/obesity.

## Introduction

The prevalence of obesity rises worldwide. This is associated with significant morbidity, costs, and decreased life expectancy. The latter can be reduced with 8-13 years^1^, which results in a huge economic burden^2^. The causes of obesity are diverse and include excessive energy intake, lack of physical activity, but also culprits such as stress, lack of sleep, or exposure to chemical endocrine disruptors^3^. There is increasing evidence from mouse as well as human studies that shows that the gut microbiome may play an important role in energy balance^4^. Modern lifestyle, and the widespread use of antibiotics may affect the composition of our microbiome, which may have consequences for our health^5^.

In this context, *Helicobacter pylori (H*. *pylori)*, is of relevance. This Gram-negative, spiral-shaped, gastric bacterium is gradually disappearing in Western populations^5,6^. *H*. *pylori* colonization is virtually always associated with chronic active gastritis, which can have various effects. This includes interference with gastric hormone regulation, including ghrelin and leptin. Both have multiple roles in energy homeostasis^7^. Disturbance of their normal regulation interferes with metabolism and our energy household. *H*. *pylori* eradication increases serum ghrelin levels^8^.

For these reasons, several epidemiological studies have focused on the correlation between *H*. *pylori* colonization and BMI and obesity. They showed contrasting results, which were based on *H*. *pylori* status and BMI data, but did not include genetic information. A recent genome-wide association study (GWAS) identified two genetic loci associated with anti-*H*. *pylori* IgG titers^9^. Numerous GWAS have identified many genetic loci associated with BMI variation and/or obesity risk^10^. Combining these results into risk scores enables a Mendelian randomization study for association between *H*. *pylori* serology and BMI. Mendelian randomization is a technique that aims at unbiased detection of causal effects^11^.

We aimed to assess the relationship between *H*. *pylori* seroprevalence and obesity using both epidemiological and genetic data of two population-based cohort studies. Results of cross-sectional and genetic analyses were compared. In addition, we performed a meta-analysis of data derived from both cohorts.

## Results

### Baseline characteristics

In total, 13,044 participants were initially included in this study. Table 1 summarizes the baseline characteristics of each cohort. In 220 subjects (1.7%) no data on BMI was available. Data on *H*. *pylori* titer was lacking in 252 individuals (1.9%) of SHIP. The total population included in the cross-sectional analyses consisted of 12,572 (96.4%) subjects. According to the predefined phenotypic seroprevalence, a total of 3,147 (25.0%) subjects were considered as cases, and 9,425 (75%) as controls.

**Table 1:** Baseline characteristics (total cohort n = 13,044).

Cohort	RS-I and RS-II	SHIP	SHIP-TREND
Total number (n)	7,977	4,081	986
Age (years), mean (sd)	69.0 (9.3)	49.7 (16.3)	50.1 (13.7)
Female sex (%)	4,391 (55.0)	2,073 (50.8)	554 (56.2)
*H*. *pylori* titer distribution, median (range)	24.3 (6.2–5587.4)	30.4 (5–500)	16.1 (5–500)
*H*. *pylori* positive (cut-off)^1^, %	4,372 (54.8)	2,275 (59.4)	440 (44.8)
*H*. *pylori* positive (highest 25% IgG titer)^2^, %	1,994 (25.0)	958 (25.0)	246 (25.0)
*H*. *pylori* antigen distribution, median (range)	NA	NA	−0.004 (−0.151, 3.983)
H. pylori antigen (cut-off)^1^, %	NA	NA	255 (26.9)
BMI (kg/m^2^), mean (sd)	26.7 (3.9)	27.3 (4.7)	27.4 (4.6)
*Missing*, *n (%)*	*209 (2.6)*	11 (0.3)	0 (0.0)
Obesity (BMI ≥ 30), %	1,343 (17.3)	1,042 (25.6)	252 (25.6)
Overweight (BMI ≥ 25), %	5,027 (64.7)	2,690 (66.1)	660 (66.9)
Obesity classes (BMI), %			
Lean, <18.5	79 (1.0)	42 (1.0)	2 (0.2)
Normal weight, 18.5–24.9	2,662 (34.3)	1,338 (32.9)	324 (32.9)
Overweight, 25.0–29.9	3,684 (47.2)	1,648 (40.5)	408 (41.4)
Class I obesity, 30.0–34.9	1,110 (14.3)	775 (19.0)	200 (20.3)
Class II obesity, 35.0–39.9	203 (2.6)	217 (5.3)	42 (4.3)
Class III obesity, ≥40.0	30 (0.4)	50 (1.2)	10 (1.0)
BMI risk score, mean (sd)	43.22 (3.98)		
*Missing*, *n (%)*	*1,094 (13.7)*	*257 (6.3)*	*3 (0.3)*
Obesity risk score, mean (sd)	42.58 (3.94)		
*Missing*, *n (%)*	*1,094 (13.7)*	*257 (6.3)*	*3 (0.3)*
*H*. *pylori* risk score, mean (sd)	3.34 (0.65)		
*Missing*, *n (%)*	*1,094 (13.7)*	*257 (6.3)*	*3 (0.3)*

^1^According to manufacturer’s definition.

^2^According to phenotype definition.

NA, not applicable.

Genotyping data was available for 6,883 (86.3%) subjects of RS, for 3,824 (93.7%) subjects of SHIP, and for 983 (99.7%) of SHIP-TREND, leaving a total population for analysis of 11,690 (89.6%) subjects. Table 1 summarizes the mean gene risk score for each cohort with respect to the correlation between BMI, obesity, and *H*. *pylori*. Linear regression analysis within RS focusing on the BMI gene score and BMI revealed a beta of 0.10 kg/m^2^ per additional BMI-increasing allele (95% CI 0.07–0.12, p = 5.29•10^−16^). Logistic regression analysis focusing on the obesity gene score and obesity (BMI ≥ 30 kg/m^2^) revealed an OR of 1.04 per additional obesity risk allele (95% CI 1.02–1.05, p = 1.09•10^−4^). The *H*. *pylori* gene score was significantly associated with *H*. *pylori* positivity (OR 1.39, 95% CI 1.27–1.51; p = 3.46•10^−13^). The proportion of variance of BMI, obesity, and *H*. *pylori* explained by the genetic risk scores ranged from 0.3% for obesity, 0.5% for *H*. *pylori* and 1% for BMI per 1 unit increase in score.

### Cross-sectional analyses

Cross-sectional analyses revealed an association between *H*. *pylori* titer and BMI in RS and SHIP (Supplementary Table S1), however with opposite direction. Meta-analysis of all three cohorts showed no association between *H*. *pylori* titer and BMI, nor between *H*. *pylori* positivity and BMI (Table 2). *H*. *pylori* titer, adjusted for age and sex, was negatively associated with obesity (OR 0.99; 95% CI 0.99–1.00, p = 0.03) and with obesity classes (Beta −6.91 •10^−5^; 95% CI −1.38•10^−4^, −5.49•10^−7^, p = 0.048) (Table 2).

**Table 2:** Cross-sectional analyses regarding serologic *H*. *pylori* status and BMI/obesity – meta-analysis.

Model	Cohort			Meta-analysis	
BMI~*H*. *pylori*	RS	SHIP	SHIP-TREND	Beta kg/m^2^ (95% CI)	p-value
*Hp* titer - crude	−	+	+	1.19•10^−3^ (−1.61•10^−3^; 3.99•10^−3^)	0.39
*Hp* titer - adjusted^1^	−	+	−	−3.09•10^−4^ (−6.46•10^−4^; 2.81•10^−5^)	0.07
*Hp* positivity – crude	−	+	+	0.37 (−0.48; 1.22)	0.40
*Hp* positivity - adjusted^1^	−	+	−	0.04 (−0.34; 0.41)	0.84
**Obesity** ^2^ **~** ***H***. ***pylori***				**OR (95% CI)**	
*Hp* titer – crude	−	+	+	1.00 (0.99; 1.00)	0.72
*Hp* titer - adjusted^1^	−	+	−	1.00 (0.99; 1.00)	**0.03**
*Hp* positivity – crude	−	+	+	1.04 (0.78; 1.39)	0.78
*Hp* positivity - adjusted^1^	−	+	−	0.95 (0.86; 1.06)	0.37
**Obesity classes** ^3^ ***~H***. ***pylori***				**Beta (95% CI)**	
*Hp* titer – crude	−	+	+	2.28•10^−4^ (−2.91•10^−4^; 7.48•10^−4^)	0.39
*Hp* titer - adjusted^1^	−	+	−	−6.91 •10^−5^ (−1.38•10^−4^; −5.49•10^−7^)	**0.05**
*Hp* positivity – crude	−	+	+	0.06 (−0.10; 0.22)	0.49
*Hp* positivity - adjusted^1^	−	+	−	−0.02 (−0.05; 0.02)	0.36

^1^Adjusted for sex and age.

^2^Obesity defined as BMI ≥ 30 kg/m^2^.

^3^Lean: BMI < 18.5 kg/m^2^; normal-weight: BMI ≥ 18.5 and <25 kg/m^2^; overweight: BMI ≥ 25 and <30 kg/m^2^; class I obesity: BMI ≥ 30 and <35 kg/m^2^; class II obesity: BMI ≥ 35 and <40 kg/m^2^, class III obesity: BMI ≥ 40 kg/m^2^.

‘−’ means a negative Beta or OR < 0.1.

‘+’ means a positive Beta or OR > 0.1.

Cross-sectional analyses regarding fecal *H*. *pylori* status and BMI/obesity showed no association (Supplementary Table S2).

### Mendelian randomization

The BMI gene score was not associated with *H*. *pylori* titer or positivity (Table 3). Supplementary Table S3 shows the results of each cohort. Crude analysis showed a positive association between obesity gene score and *H*. *pylori* titer (Beta 0.76; 95% CI 0.02–1.50, p = 0.04) (Table 3). *H*. *pylori* gene score was not associated with BMI, neither with obesity nor obesity classes (Table 4 and Supplementary Table S4). Also, no associations were observed regarding fecal *H*. *pylori* status and the BMI or obesity gene score (Supplementary Table S5).

**Table 3:** Mendelian randomization *H*. *pylori* and BMI/obesity gene score – meta-analysis.

Model	Cohort			Meta-analysis	
*H*. *pylori* titer~BMI gene score	RS	SHIP	SHIP-TREND	Beta (95% CI)	p-value
BMI gene score – crude	+	+	+	0.12 (−0.63; 0.87)	0.76
BMI gene score – adjusted^1^	+	−	+	0.03 (−0.71; 0.77)	0.95
BMI gene score – adjusted^2^	+	−	+	0.04 (−0.71; 0.78)	0.92
***H***. ***pylori*** **positivity~BMI gene score**				**OR (95% CI)**	
BMI gene score – crude	+	+	−	1.01 (1.00; 1.02)	0.14
BMI gene score – adjusted^1^	+	+	−	1.01 (1.00; 1,02)	0.20
BMI gene score – adjusted^2^	+	+	−	1.01 (1.00; 1,02)	0.15
***H***. ***pylori*** **titer~Obesity gene score**				**Beta (95% CI)**	
Obesity gene score – crude	+	+	−	0.76 (0.02; 1.50)	**0.04**
Obesity gene score – adjusted^1^	+	+	−	0.73 (−0.00; 1.47)	0.05
Obesity gene score – adjusted^2^	+	+	−	0.71 (−0.03; 1.45)	0.06
***H***. ***pylori*** **positivity~Obesity gene score**				**OR (95% CI)**	
Obesity gene score – crude	+	+	−	1.01 (1.00; 1.02)	0.12
Obesity gene score – adjusted^1^	+	+	−	1.01 (1.00; 1.02)	0.14
Obesity gene score – adjusted^2^	+	+	−	1.01 (1.00; 1.02)	0.15

^1^Adjusted for age and sex.

^2^Adjusted for age, sex, and BMI.

‘−’ means a negative Beta or OR < 0.1.

‘+’ means a positive Beta or OR > 0.1.

**Table 4:** Mendelian randomization regarding BMI/obesity and *H*. *pylori* gene score – meta-analysis.

Model	Cohort			Meta-analysis	
BMI~*H*. *pylori* gene score	RS	SHIP	SHIP-TREND	Beta (95% CI)	p-value
*Hp* gene score – crude	−	−	−	−0.05 (−0.16; 0.07)	0.43
*Hp* gene score – adjusted^1^	−	−	−	−0.06 (−0.17; 0.05)	0.31
*Hp* gene score – adjusted^2^	−	−	−	−0.05 (−0.17; 0.06)	0.37
**Obesity3~** ***H***. ***pylori*** **gene score**				**OR (95% CI)**	
*Hp* gene score – crude	−	+	−	0.98 (0.92; 1.05)	0.60
*Hp* gene score – adjusted^1^	−	−	−	0.98 (0.91; 1.04)	0.47
*Hp* gene score – adjusted^2^	+	−	−	0.98 (0.91; 1.05)	0.56
**Obesity classes** ^4^~***H***. ***pylori*** **gene score**				**Beta (95% CI)**	
*Hp* gene score – crude	+	−	−	−0.01 (−0.03; 0.02)	0.54
*Hp* gene score – adjusted^1^	+	−	−	−0.01 (−0.03; 0.01)	0.37
*Hp* gene score – adjusted^2^	+	−	−	−0.03 (−0.06; 0.01)	0.15

^1^Adjusted for age and sex.

^2^Adjusted for age, sex, and *H*. *pylori*.

^3^Obesity defined as BMI ≥ 30 kg/m^2^. ^4^Obesity classes defined as lean: BMI < 18.5 kg/m^2^; normal-weight: BMI ≥ 18.5 and <25 kg/m^2^; overweight: BMI ≥ 25 and <30 kg/m^2^; class I obesity: BMI ≥ 30 and <35 kg/m^2^; class II obesity: BMI ≥ 35 and <40 kg/m^2^, class III obesity: BMI ≥ 40 kg/m^2^.

‘−’ means a negative Beta or OR < 0.1.

‘+’ means a positive Beta or OR > 0.1.

## Discussion

This study included a meta-analysis of 13,044 subjects from two large population-based cohorts. This analysis did not demonstrate an association between *H*. *pylori* colonization and BMI, neither when examined by means of serology, nor by fecal antigen, or Mendelian randomization. *H*. *pylori* serology, adjusted for age and sex, was negatively associated with obesity (BMI ≥ 30 kg/m^2^), and obesity classes. However, these effects were small. Active *H*. *pylori* colonization, determined by a positive fecal antigen test, was also not positively or negatively associated with obesity. While the unadjusted and adjusted effect estimates for the obesity gene score on anti-*H*. *pylori* IgG titer were positive, this association did not remain statistically significant after adjustment for age and sex. So, the use of a Mendelian randomization method did not show a causal bi-directional link between *H*. *pylori* serology and BMI or obesity.

Our meta-analysis of *H*. *pylori* status as determined by serology showed a small negative association with both obesity and obesity classes. Considering both the small effect estimates, and opposite directions in the individual cohorts, we consider these associations as clinically irrelevant. Prior epidemiological studies have shown either negative^12^, or positive^13,14^, or no association^15,16^ between *H*. *pylori* and BMI or obesity. The latter findings are most in line with our findings. A recent review of studies reporting data on *H*. *pylori* and obesity prevalence rates in developed countries, showed an inverse correlation (r = −0.29, p < 0.001) between *H*. *pylori* colonization and obesity and overweight^17^. In total, data of 99,463 subjects from 49 studies were pooled. Prevalence rates for *H*. *pylori*, but also for overweight and obesity were highly variable between included studies. Nevertheless, no additional analyses were performed to examine whether this correlation was related to potential significant confounders such as age. Age is an important confounder as it is positively correlated with *H*. *pylori* colonization^18^, and negatively with obesity^18^. This may explain the negative correlation between *H*. *pylori* and obesity reported in the systematic review. Other studies have observed weight gain following successful *H*. *pylori* eradication^19–23^. A clinical trial from Japan randomized 1,558 *H*. *pylori*-positive adults to either antibiotic treatment or placebo with a subsequent follow-up of 6 months. *H*. *pylori* eradication was associated with a mean weight gain of 0.6 kg (95% CI 0.31, 0.88) and an increase in BMI of 0.2 kg/m^2^ ^19^. The simultaneous improvement of dyspepsia symptoms in the eradication group may have stimulated the appetite and subsequently caused the weight gain by increased food intake. Others have suggested that circulating meal-associated leptin and ghrelin levels, which changed after *H*. *pylori* eradication, gave rise to the increased BMI. US investigators observed an increase in both post-prandial levels of leptin and ghrelin a median seven months following *H*. *pylori* eradication in a group of 21 patients^24^. In addition, BMI significantly increased after over 18 months of follow-up, while no change was observed in those who were *H*. *pylori-*negative at baseline. Although these studies provided evidence that *H*. *pylori* eradication may result in weight gain, it does not imply that there is an absolute difference in BMI between *H*. *pylori*-negative and positive subjects. Both our cross-sectional as well as Mendelian randomization results suggest that *H*. *pylori*-colonized and *H*. *pylori*-negative subjects have similar BMI. The congruence between the results of our cross-sectional and Mendelian randomization analyses is important, as the latter is based on an unbiased approach^11^.

One of the strengths of this study was the use of different methods to detect *H*. *pylori* colonization, by means of both serology and stool antigen. The latter is a reliable method to identify active *H*. *pylori* colonization. While most prior studies reported data on serology, we were able to show that results for serology and fecal antigen did not differ. In addition, the use of SNP typing data with genetic risk scores for BMI, obesity, and *H*. *pylori* colonization is unique in this field. The Mendelian randomization method is a powerful control for reverse causation and confounding, which otherwise affects epidemiological studies^11^. It is based on the common disease, common variant hypothesis, which argues that common variants with modest effects underlie many complex traits^25^.

As any method, the Mendelian randomization has its limitations. Although many genetic variants are discovered, these common variants only explain a small proportion of the estimated trait heritability. Regarding the *H*. *pylori*-gene risk score, we were only able to include two genetic variants, due to the fact that no others (as far as we know) have been discovered so far, and these explain only 0.5% of the variance. Similarly, a recent large-scale consortium study estimates that 97 GWAS loci account for ∼2.7% of BMI variation^26^. Data on *H*. *pylori* eradication was not available in the RS and SHIP cohort. Nevertheless, given the selective indications for this both in The Netherlands and in Germany, we can safely assume that this was a small minority of the total population. Finally, we did not account for socio-economic status in the cross-sectional analyses. Although both populations are similar regarding ethnicity and age distribution, differences in socio-economic status are associated with both *H*. *pylori* colonization and BMI, and may therefore have influenced our outcome.

In conclusion, this study provides no evidence for a cross-sectional association between *H*. *pylori* colonization and BMI or obesity in adults. Mendelian randomization revealed no causal relation between *H*. *pylori* and BMI or obesity.

## Methods

### Study cohorts

The Rotterdam Study is a large, population based prospective study of elderly individuals of European ancestry consisting of three cohorts (RS-I, RS-II, RS-III), who are residing in a suburb of Rotterdam, the Netherlands. The study design has been described in detail previously^23,27^. Baseline recruitment and measurements for the RS-I study were obtained between 1990 and 1993. The second cohort, RS-II, was set-up in 2000–2001. A third cohort, RS-III, started in 2006 and recruitment ended in December 2008.

The SHIP study comprises two independent prospectively recruited population-based cohorts in Northeastern Germany: SHIP and SHIP-TREND. The study design of SHIP has been described in detail previously^18^. Participants were recruited between October 1997 and May 2001. SHIP-TREND is an independent cohort from the same region. Individuals were recruited between September 2008 and summer 2012^18^. An important characteristic of SHIP is that it attempts to describe health-related conditions with the widest focus possible.

Data from SHIP, SHIP-TREND, RS-I, and RS-II (RS from now on) were used in this study. Written informed consent was obtained from all participants. Both the medical ethics committee of the Erasmus MC University Medical Center Rotterdam and University Medicine Greifswald approved the study. All methods were performed in accordance with the relevant guidelines and regulations, approved by the medical ethics committee.

### Phenotype definition

Serologic *H*. *pylori* colonization in individuals from SHIP, SHIP-TREND, and RS was defined by measuring IgG antibody levels in serum using commercial enzyme-linked immunosorbent assay (Pyloriset EIA-G III ELISA; Orion). Seropositivity was defined as an anti-*H*. *pylori* IgG titer of ≥20 U/mL according to the manufacturer’s instructions^28^. Seropositivity is an indicator for current or past colonization. The sensitivity of the Pyloriset EIA-G III ELISA is reported as 97.8%, with a specificity of 58%^29^. To increase specificity and reduce the number of false-positive *H*. *pylori* infections, we defined subjects with the 25% highest IgG titers as *H*. *pylori* cases, and those with the 75% lowest IgG titers as controls^9^.

We further assessed the presence of *H*. *pylori* antigen in stool of subjects from SHIP-TREND by using the *H*. *pylori* antigen ELISA kit (Immunodiagnostics). For this purpose, 100 mg feces was stored at −20 °C. An optical density (OD) ≥ 0.025 at 450 nm was considered evidence of *H*. *pylori* infection, according to the manufacturer’s recommendation. This test has a sensitivity of 97.7% and specificity of 96.3%. *H*. *pylori* stool antigen levels and measured anti-*H*. *pylori* IgG titers are positively correlated (Spearman ρ = 0.59, p < 0.001)^9^.

### Genetic risk score conduction

For the creation of the genetic risk scores (BMI risk score, obesity risk score, *H*. *pylori* risk score), we firstly searched the literature for publications of genome-wide association studies (GWAS) for these traits. A list of SNPs that reached genome-wide significance (P < 5 × 10^−8^) with BMI or binary obesity status in populations of European ancestry was established. Three different strategies were used to optimize the SNP selection procedure using a key word search (e.g. BMI) on i) the National Human Genome Research Institute (NHGRI) GWAS Catalog (www.genome.gov/gwastudies/) ii) the HuGE Navigator GWAS Integrator (www.hugenavigator.net/HuGENavigator/gWAHitStartPage.do) iii) the PubMed database (www.ncbi.nlm.nih.gov/pubmed). Using this strategy, 45 independent loci were found to be associated with BMI variation and 48 with binary obesity status. We chose to analyze risk scores for BMI and obesity separately. BMI is a phenotype which results in a relatively clean risk score. In contrast, various different definitions have been used to define obesity, like BMI ≥ 25, or BMI ≥ 30. For this reason we used both phenotypes (e.g. BMI continuous and binary) in our analyses. A genotype score (GS) was calculated by summing the alleles of BMI / obesity / *H*. *pylori* status-associated SNPs. An unweighted GS was used as previously recommended by Dudbridge *et al*.^30^. Imputed genotypes (1000 G PhaseIv3) were used for the creation of the GS. All GWAS published before June 2014 were included. Per genetic locus, 1 SNP was selected based on the following criteria: 1) SNP genotyped with an imputation quality (R2) of 0.3 or higher in all populations; 2) Preference for A/C or G/T variants to avoid strand issues. A list of all studies and variants considered, as well as the variants selected can be found in Supplementary Tables S6, S7 and S8.

### Statistical analysis

In total, we used three different approaches to assess the relationship between *H*. *pylori* status and BMI/obesity. First, cross-sectional analyses were performed to assess the relationship between *H*. *pylori* colonization and BMI/obesity at time of inclusion, by using linear and logistic regression. Outcomes were defined as continuous BMI, binary obesity (BMI ≥ 30 kg/m^2^, with BMI < 30 kg/m^2^ as reference group), and obesity classes (lean BMI < 18.5 kg/m^2^; normal-weight BMI ≥ 18.5 and <25 kg/m^2^; overweight BMI ≥ 25 and <30 kg/m^2^; class I obesity BMI ≥ 30 and <35 kg/m^2^; class II obesity BMI ≥ 35 and <40 kg/m^2^, class III obesity BMI ≥ 40 kg/m^2^). The latter outcome was defined as a continuous variable with value ‘0’ for lean, and value ‘5’ for class III obesity. Unadjusted effects of *H*. *pylori* titer or antigen (continuous) and *H*. *pylori* positivity (binary phenotype) were assessed for each outcome. We additionally adjusted for sex and age. All analyses were done separately for RS, SHIP, and SHIP-TREND. A meta-analysis was performed to observe the combined effect of *H*. *pylori* colonization on BMI/obesity.

Second, a Mendelian randomization approach was carried out to explore a bi-directional link between *H*. *pylori* colonization and BMI. Linear regression analysis was used to assess the relationship between BMI gene score and *H*. *pylori* titer and *H*. *pylori* positivity. Analyses were first adjusted for sex, age, and additionally for BMI at baseline. The same analyses were done regarding the obesity gene score. Linear regression analysis was also used to examine the effect of the *H*. *pylori* gene score with BMI, binary obesity status, and obesity classes, all defined at baseline. Analyses were adjusted for sex, and age, and additionally for *H*. *pylori* status (binary phenotype). Data of the three cohorts were combined and examined by using meta-analysis approach. A p-value < 0.05 was considered to be statistically significant.

All measures of associations are presented as Odds Ratios (OR) or Beta’s with their 95% confidence intervals (CI). Statistical analyses were performed using IBM SPSS Statistics 21.0 for Windows (SPSS IBM, Armonk, New York, USA). Meta-analyses were done with R library meta (R Core Team (2014), R Foundation for Statistical Computing, Vienna, Austria).

### Data availability

Due to ethical restrictions data are available upon request. Interested researchers may contact our data management team (secretariat.epi@erasmusmc.nl) for access to sensitive data.

## Electronic supplementary material

Supplemantary data
