# Supplementary material for: Helicobacter pylori colonization and obesity – a Mendelian randomization study
Source: Sci Rep. 2017 Oct 31;7:14467. doi: 10.1038/s41598-017-14106-4 (PMC5663904; doi:10.1038/s41598-017-14106-4)
Supplement: Supplementary file 1 — Supplemantary data [file 41598_2017_14106_MOESM1_ESM.pdf]

**Supplementary data**

# *Helicobacter pylori* colonization and obesity – a Mendelian randomization study

Wouter J. den Hollander[1*], Linda Broer[2*], Claudia Schurmann[3,4], David Meyre[5,6], Caroline M. den Hoed[1], Julia Mayerle[7], Albert Hofman[8,10], Georg Homuth[9], André G. Uitterlinden[2,8], Markus M. Lerch[7], Ernst J. Kuipers[1, 2]

[1]Department of Gastroenterology and Hepatology, [2]Internal Medicine, and [8]Epidemiology, Erasmus MC University Medical Centre, Rotterdam, The Netherlands; [3]The Charles Bronfman Institute for Personalized Medicine, and [4]The Genetics of Obesity and Related Metabolic Traits Program, Icahn School of Medicine at Mount Sinai, New York, NY, USA; [5]Department of Health Research Methods, Evidence, and Impact, and [6]Department of Pathology and Molecular Medicine, McMaster University, Hamilton, Canada; [7]Department of Medicine A, and [9]Interfaculty Institute for Genetics and Functional Genomics, University Medicine Greifswald, Germany; [10]Department of Epidemiology, Harvard T.H. Chan School of Public Health, Boston, MA, USA.

[*]Shared first authorship

**Supplementary Table S1 Cross-sectional analyses regarding serologic *H. pylori* status and BMI/obesity**

| | Cohort | | | | | | | | |
|---|---|---|---|---|---|---|---|---|---|
| **Model** | **RS** | | | **SHIP** | | | **SHIP-TREND** | | |
| **BMI~*H. pylori*** | **Beta** | **se** | **p-value** | **Beta** | **se** | **p-value** | **Beta** | **se** | **p-value** |
| *Hp* titer - crude | $-4.93 \cdot 10^{-4}$ | $1.83 \cdot 10^{-4}$ | **0.01** | $3.30 \cdot 10^{-3}$ | $6.31 \cdot 10^{-4}$ | **$2.51 \cdot 10^{-7}$** | $8.06 \cdot 10^{-4}$ | $1.16 \cdot 10^{-3}$ | 0.49 |
| *Hp* titer - adjusted[1] | $-4.27 \cdot 10^{-4}$ | $1.82 \cdot 10^{-4}$ | **0.02** | $0.95 \cdot 10^{-3}$ | $6.11 \cdot 10^{-4}$ | 0.12 | $-4.19 \cdot 10^{-4}$ | $1.12 \cdot 10^{-3}$ | 0.71 |
| *Hp* positivity - crude | -0.22 | 0.10 | **0.03** | 0.96 | 0.18 | **$6.36 \cdot 10^{-8}$** | 0.39 | 0.34 | 0.24 |
| *Hp* positivity - adjusted[1] | -0.18 | 0.10 | 0.07 | 0.33 | 0.17 | 0.05 | $-8.22 \cdot 10^{-3}$ | 0.33 | 0.98 |
| **Obesity[2]~*H. pylori*** | | | | | | | | | |
| *Hp* titer - crude | $-4.09 \cdot 10^{-4}$ | $1.47 \cdot 10^{-4}$ | **0.01** | $7.20 \cdot 10^{-4}$ | $2.95 \cdot 10^{-4}$ | **0.01** | $3.16 \cdot 10^{-4}$ | $5.70 \cdot 10^{-4}$ | 0.58 |
| *Hp* titer - adjusted[1] | $-3.92 \cdot 10^{-4}$ | $1.48 \cdot 10^{-4}$ | **0.01** | $0.90 \cdot 10^{-4}$ | $3.07 \cdot 10^{-4}$ | 0.77 | $-9.55 \cdot 10^{-6}$ | $5.83 \cdot 10^{-4}$ | 0.99 |
| *Hp* positivity - crude | -0.15 | 0.07 | **0.04** | 0.26 | 0.08 | **$2.03 \cdot 10^{-3}$** | $1.25 \cdot 10^{-2}$ | 0.17 | 0.94 |
| *Hp* positivity - adjusted[1] | -0.13 | 0.07 | 0.07 | 0.08 | 0.09 | 0.31 | $-9.57 \cdot 10^{-2}$ | 0.17 | 0.58 |
| **Obesity classes[3]~*H. pylori*** | | | | | | | | | |
| *Hp* titer - crude | $-1.04 \cdot 10^{-4}$ | $3.80 \cdot 10^{-5}$ | **0.01** | $5.62 \cdot 10^{-4}$ | $1.25 \cdot 10^{-4}$ | **$7.32 \cdot 10^{-6}$** | $1.78 \cdot 10^{-4}$ | $2.27 \cdot 10^{-4}$ | 0.43 |
| *Hp* titer - adjusted[1] | $-9.24 \cdot 10^{-5}$ | $3.80 \cdot 10^{-5}$ | **0.01** | $1.49 \cdot 10^{-4}$ | $1.23 \cdot 10^{-4}$ | 0.23 | $-5.52 \cdot 10^{-5}$ | $2.21 \cdot 10^{-4}$ | 0.80 |
| *Hp* positivity - crude | -0.05 | 0.02 | **0.02** | 0.17 | 0.04 | **$1.13 \cdot 10^{-6}$** | 0.05 | 0.07 | 0.45 |
| *Hp* positivity - adjusted[1] | -0.04 | 0.02 | **0.04** | 0.06 | 0.03 | 0.08 | -0.03 | 0.06 | 0.69 |

[1]adjusted for sex and age

[2]Obesity defined as BMI>30

[3] Lean (BMI < 18.5); normal-weight BMI >= 18.5 and < 25; overweight BMI >=25 and < 30; class I obesity BMI >=30 and < 35; class II obesity BMI >= 35 and < 40, class III obesity BMI >= 40

**Supplementary Table S2. Cross-sectional analyses regarding fecal *H. pylori* status and BMI/obesity**

| Model | SHIP-TREND | |
|---|---|---|
| **BMI~*H. pylori*** | **Beta (95% CI)** | **p-value** |
| *Hp* titer - crude | 0.12 (-0.24, 0.48) | 0.51 |
| *Hp* titer - adjusted[1] | -0.08 (-0.43, 0.27) | 0.67 |
| *Hp* positivity - crude | 0.21 (-0.44; 0.86) | 0.52 |
| *Hp* positivity - adjusted[1] | -0.05 (-0.68; 0.58) | 0.88 |
| **Obesity[2]~*H. pylori*** | **OR (95% CI)** | |
| *Hp* titer - crude | 1.00 (0.83; 1.19) | 0.97 |
| *Hp* titer - adjusted[1] | 0.94 (0.78; 1.13) | 0.52 |
| *Hp* positivity - crude | 0.95 (0.68; 1.32) | 0.76 |
| *Hp* positivity - adjusted[1] | 0.88 (0.63; 1.23) | 0.46 |
| **Obesity classes[3]~*H. pylori*** | **Beta (95% CI)** | |
| *Hp* titer - crude | 0.01 (-0.04; 0.08) | 0.82 |
| *Hp* titer - adjusted[1] | -0.03 (-0.01; 0.04) | 0.39 |
| *Hp* positivity - crude | 0.02 (-0.13; 0.15) | 0.81 |
| *Hp* positivity - adjusted[1] | -0.04 (-0.17; 0.09) | 0.59 |

[1]Adjusted for age and sex

[2]Obesity defined as BMI>30

[3]Lean (BMI < 18.5); normal-weight BMI >= 18.5 and < 25; overweight BMI >=25 and < 30; class I obesity BMI >=30 and < 35; class II obesity BMI >= 35 and < 40, class III obesity BMI >= 40

**Supplementary Table S3 Mendelian randomization regarding serologic *H. pylori* status and BMI/obesity gene score**

| | Cohort | | | | | | | | |
|---|---|---|---|---|---|---|---|---|---|
| **Model** | **RS** | | | **SHIP** | | | **SHIP-TREND** | | |
| ***H. pylori* titer~BMI gene score** | **Beta** | **se** | **p-value** | **Beta** | **se** | **p-value** | **Beta** | **se** | **p-value** |
| BMI gene score – crude | 0.18 | 0.73 | 0.80 | 0.03 | 0.49 | 0.95 | 0.41 | 1.08 | 0.70 |
| BMI gene score – adjusted[1] | 0.16 | 0.73 | 0.83 | -0.06 | 0.48 | 0.90 | 0.17 | 1.08 | 0.87 |
| BMI gene score – adjusted[2] | 0.49 | 0.74 | 0.51 | -0.18 | 0.48 | 0.70 | 0.20 | 1.08 | 0.85 |
| ***H. pylori* positivity~BMI gene score** | | | | | | | | | |
| BMI gene score – crude | 0.01 | 0.01 | 0.14 | 0.01 | 0.01 | 0.46 | $-3.73 \cdot 10^{-3}$ | 0.02 | 0.85 |
| BMI gene score – adjusted[1] | 0.01 | 0.01 | 0.15 | 0.01 | 0.01 | 0.58 | -0.01 | 0.02 | 0.62 |
| BMI gene score – adjusted[2] | 0.01 | 0.01 | 0.06 | $2.18 \cdot 10^{-3}$ | 0.01 | 0.82 | -0.01 | 0.02 | 0.62 |
| ***H. pylori* titer~Obesity gene score** | | | | | | | | | |
| Obesity gene score – crude | 0.77 | 0.74 | 0.30 | 1.01 | 0.49 | **0.04** | -0.43 | 1.06 | 0.69 |
| Obesity gene score – adjusted[1] | 0.76 | 0.74 | 0.31 | 0.97 | 0.48 | **0.04** | -0.49 | 1.05 | 0.64 |
| Obesity gene score – adjusted[2] | 1.05 | 0.75 | 0.16 | 0.81 | 0.48 | 0.09 | -0.46 | 1.06 | 0.66 |
| ***H. pylori* positivity~Obesity gene score** | | | | | | | | | |

| | | | | | | | | | |
|---|---|---|---|---|---|---|---|---|---|
| Obesity gene score – crude | 0.01 | 0.01 | 0.42 | 0.02 | 0.01 | 0.11 | $-4.13 \cdot 10^{-3}$ | 0.02 | 0.83 |
| Obesity gene score – adjusted[1] | 0.01 | 0.01 | 0.43 | 0.01 | 0.01 | 0.12 | $-5.64 \cdot 10^{-3}$ | 0.02 | 0.77 |
| Obesity gene score – adjusted[2] | 0.01 | 0.01 | 0.26 | 0.01 | 0.01 | 0.26 | $-5.71 \cdot 10^{-3}$ | 0.02 | 0.77 |

[1]Adjusted for age and sex

[2]Adjusted for age, sex, and BMI

**Supplementary Table S4 Mendelian randomization regarding BMI/obesity and *H. pylori* gene score**

| | Cohort | | | | | | | | |
|---|---|---|---|---|---|---|---|---|---|
| **Model** | **RS** | | | **SHIP** | | | **SHIP-TREND** | | |
| **BMI~*H. pylori* gene score** | **Beta** | **se** | **p-value** | **Beta** | **se** | **p-value** | **Beta** | **se** | **p-value** |
| *Hp* gene score – crude | -0.02 | 0.07 | 0.74 | -0.04 | 0.11 | 0.69 | -0.24 | 0.22 | 0.26 |
| *Hp* gene score – adjusted[1] | -0.02 | 0.07 | 0.76 | -0.08 | 0.11 | 0.43 | -0.28 | 0.21 | 0.18 |
| *Hp* gene score – adjusted[2] | -0.01 | 0.07 | 0.92 | -0.09 | 0.11 | 0.40 | -0.28 | 0.21 | 0.19 |
| **Obesity[3]~*H. pylori* gene score** | | | | | | | | | |
| *Hp* gene score – crude | $-1.00 \bullet 10^{-3}$ | 0.05 | 0.99 | $3.35 \bullet 10^{-3}$ | 0.05 | 0.95 | -0.18 | 0.11 | 0.09 |
| *Hp* gene score – adjusted[1] | $-3.46 \bullet 10^{-4}$ | 0.05 | 1.00 | $-9.67 \bullet 10^{-3}$ | 0.05 | 0.86 | -0.19 | 0.11 | 0.07 |
| *Hp* gene score – adjusted[2] | 0.01 | 0.05 | 0.87 | $-7.23 \bullet 10^{-3}$ | 0.06 | 0.90 | -0.20 | 0.11 | 0.06 |
| **Obesity classes[4]~*H. pylori* gene score** | | | | | | | | | |
| *Hp* gene score – crude | $2.00 \bullet 10^{-3}$ | 0.02 | 0.92 | -0.01 | 0.02 | 0.53 | -0.06 | 0.04 | 0.17 |
| *Hp* gene score – adjusted[1] | $2.00 \bullet 10^{-3}$ | 0.02 | 0.89 | -0.02 | 0.02 | 0.33 | -0.06 | 0.04 | 0.12 |
| *Hp* gene score – adjusted[2] | $5.00 \bullet 10^{-3}$ | 0.05 | 0.87 | -0.02 | 0.02 | 0.34 | -0.06 | 0.04 | 0.12 |

[1]Adjusted for age and sex

[2]Adjusted for age, sex, and *H. pylori*

[3]Obesity defined as BMI>30

[4]Obesity classes defined as lean (BMI < 18.5); normal-weight BMI >= 18.5 and < 25; overweight BMI >=25 and < 30; class I obesity BMI >=30 and < 35; class II obesity BMI >= 35 and < 40, class III obesity BMI >= 40

**Supplementary Table S5. Cross-sectional analyses regarding fecal *H. pylori* status and BMI/obesity gene score**

| **Model** | **Cohort SHIP-TREND** | |
|---|---|---|
| ***H. pylori* titer~BMI gene score** | **Beta (95% CI)** | **p-value** |
| BMI gene score – crude | $-1.20\cdot10^{-3}$ ($-1.5\cdot10^{-2}$; $1.27\cdot10^{-2}$) | 0.86 |
| BMI gene score – adjusted[1] | $-2.20\cdot10^{-3}$ ($-1.59\cdot10^{-2}$; $1.59\cdot10^{-2}$) | 0.75 |
| BMI gene score – adjusted[2] | $-2.10\cdot10^{-3}$ ($-1.58\cdot10^{-2}$; $1.16\cdot10^{-2}$) | 0.77 |
| ***H. pylori* positivity~BMI gene score** | **OR (95% CI)** | |
| BMI gene score – crude | 1.00 (0.99; 1.01) | 1.00 |
| BMI gene score – adjusted[1] | 1.00 (0.99; 1.01) | 0.88 |
| BMI gene score – adjusted[2] | 1.00 (0.99; 1.01) | 0.89 |
| ***H. pylori* titer~Obesity gene score** | **Beta (95% CI)** | |
| Obesity gene score – crude | $2.60\cdot10^{-3}$ ($-1.11\cdot10^{-2}$; $1.61\cdot10^{-2}$) | 0.70 |
| Obesity gene score – adjusted[1] | $2.20\cdot10^{-3}$ ($-1.11\cdot10^{-2}$; $1.55\cdot10^{-2}$) | 0.75 |
| Obesity gene score – adjusted[2] | $2.40\cdot10^{-3}$ ($-1.09\cdot10^{-2}$; $1.57\cdot10^{-2}$) | 0.72 |
| ***H. pylori* positivity~Obesity gene score** | **OR (95% CI)** | |
| Obesity gene score – crude | 1.00 (0.99; 1.01) | 0.99 |
| Obesity gene score – adjusted[1] | 1.00 (0.99; 1.01) | 0.99 |
| Obesity gene score – adjusted[2] | 1.00 (0.99; 1.01) | 0.99 |

[1]Adjusted for age and sex

[2]Adjusted for age, sex, and BMI

**Supplementary Table S6. List of SNPs associated with BMI or obesity**

| BMI | | | |
|---|---|---|---|
| **Gene** | **RS number** | **Trait** | **Reference** |
| FTO | rs9939609, rs9930506, rs1121980, rs1421085, rs8050136, rs1558902, rs17817449, rs12149832, rs9940128, rs62033400 | BMI | Frayling Science 2007, Scuteri PLOS Genet 2007, Loos Nat Genet 2008, Thorleifsson Nat Genet 2009, Willer Nat Genet 2009, Cho Nat Genet 2009, Speliotes Nat Genet 2010, Wen Nat Genet 2012, Okada Nat Genet 2012, Guo HMG 2012, Graff Hum Mol Genet 2013, Pei HMG 2013 |
| MC4R | rs17782313, rs571312, rs12970134, rs2331841, rs6567160, rs8089364, rs7234864, rs723486 | BMI | Loos Nat Genet 2008, Thorleifsson Nat Genet 2009, Speliotes Nat Genet 2010, Paternoster PLOS One 2011Okada Nat Genet 2012, Wen Nat Genet 2012, Graff Hum Mol Genet 2013, Pei HMG 2013 |
| MC4R | rs7227255, rs2229616 | BMI | Speliotes Nat Genet 2010, Guo HMG 2012 |
| TMEM18 | rs6548238, rs7561317, rs2867125, rs12463617 | BMI | Willer Nat Genet 2009, Thorleifsson Nat Genet 2009, Speliotes Nat Genet 2010, Guo HMG 2012, Graff Hum Mol Genet 2013 |
| GNPDA2 | rs10938397, rs13130484, rs348495 | BMI | Willer Nat Genet 2009, Speliotes Nat Genet 2010, Graff Hum Mol Genet 2013 |
| SH2B1 | rs7498665, rs4788102, rs7359397, rs4788099 | BMI | Willer Nat Genet 2009, Thorleifsson Nat Genet 2009, Speliotes Nat Genet 2010, Guo HMG 2012 |
| KCTD15 | rs11084753, rs29941 | BMI | Willer Nat Genet 2009, Thorleifsson Nat Genet 2009, Speliotes Nat Genet 2010 |
| MTCH2 | rs10838738, rs3817334 | BMI | Willer Nat Genet 2009, Speliotes Nat Genet 2010 |
| NEGR1 | rs2815752, rs2568958 | BMI | Willer Nat Genet 2009, Thorleifsson Nat Genet 2009, Speliotes Nat Genet 2010 |
| SEC16B | rs10913469, rs543874, rs574367, rs516636, rs591120 | BMI | Thorleifsson Nat Genet 2009, Speliotes Nat Genet 2010, Graff Hum Mol Genet 2013, Wen Nat Genet 2012, Okada Nat Genet 2012 |
| ETV5 | rs7647305, rs9816226 | BMI | Thorleifsson Nat Genet 2009, Speliotes Nat Genet 2010 |
| BDNF | rs6265, rs4923461, rs10767664, rs2030323, rs10767664 | BMI | Thorleifsson Nat Genet 2009, Speliotes Nat Genet 2010, Wen Nat Genet 2012, Okada Nat Genet 2012, Guo HMG 2012 |
| FAIM2 | rs7138803, rs7132908 | BMI | Thorleifsson Nat Genet 2009, Speliotes Nat Genet 2010, Paternoster PLOS One 2011 |
| TFAP2B | rs987237, rs734597, rs2272903 | BMI | Speliotes Nat Genet 2010, Paternoster PLOS One 2011, Guo HMG 2012 |
| NRXN3 | rs10150332 | BMI | Speliotes Nat Genet 2010 |
| GPRC5BB | rs12444979 | BMI | Speliotes Nat Genet 2010 |
| POMC | rs713586, rs6545814, rs1561288 | BMI | Speliotes Nat Genet 2010, Wen Nat Genet 2012, Graff Hum Mol Genet 2013 |
| MAP2K5 | rs2241423, rs4776970, rs997295 | BMI | Speliotes Nat Genet 2010, Wen Nat Genet 2012, Guo HMG 2012 |
| GIPR | rs2287019, rs11671664 | BMI | Speliotes Nat Genet 2010, Wen Nat Genet 2012, Okada Nat Genet 2012 |
| FANCL | rs887912 | BMI | Speliotes Nat Genet 2010 |
| TNNI3K | rs1514175, rs12142020 | BMI | Speliotes Nat Genet 2010, Graff Hum Mol Genet 2013 |
| LRRN6C | rs10968576 | BMI | Speliotes Nat Genet 2010 |
| FLJ35779 | rs2112347 | BMI | Speliotes Nat Genet 2010 |
| SLC39A8 | rs13107325 | BMI | Speliotes Nat Genet 2010 |
| TMEM160 | rs3810291 | BMI | Speliotes Nat Genet 2010 |
| CADM2 | rs13078807 | BMI | Speliotes Nat Genet 2010 |
| LRP1B | rs2890652 | BMI | Speliotes Nat Genet 2010 |
| PRKD1 | rs11847697 | BMI | Speliotes Nat Genet 2010 |

| | | | |
|---|---|---|---|
| MTIF3 | rs4771122 | BMI | Speliotes Nat Genet 2010 |
| ZNF608 | rs48361333 | BMI | Speliotes Nat Genet 2010 |
| PTBP2 | rs1555543 | BMI | Speliotes Nat Genet 2010 |
| TUB | rs4929949 | BMI | Speliotes Nat Genet 2010 |
| HMGA1 | rs206936 | BMI | Speliotes Nat Genet 2010 |
| CDKAL1 | rs2206734, rs9356744 | BMI | Wen Nat Genet 2012, Okada Nat Genet 2012 |
| PCSK1 | rs261967 | BMI | Wen Nat Genet 2012 |
| GP2 | rs12597579 | BMI | Wen Nat Genet 2012 |
| KLF9 | rs11142387 | BMI | Okada Nat Genet 2012 |
| TOMM40/APOE/APOC1 | rs2075650 | BMI | Guo HMG 2012 |
| FANCL/FLJ30838 | rs12617233 | BMI | Guo HMG 2012 |
| NTRK2 | rs1211166 | BMI | Guo HMG 2012 |
| GALNT10 | rs7708584 | BMI | Monda NG 2013 african |
| MIR148A/NFE2L3 | rs10261878 | BMI | Monda NG 2013 african + european |
| ADCY3 | rs7586879, rs6545814 | BMI | Wen Nat Genet 2012 east asian, Monda NG 2013 african |
| BRE | rs116612809 | BMI | Gong AJHG 2013 |
| MAP2K3 | rs11652094 | BMI | Bian Hum Mol Genet 2013 Pimas + Europeans |

## Obesity binary status

| Gene | RS number | Trait | Reference |
|---|---|---|---|
| FTO | rs1421085, rs1121980, rs9936385, rs9941349, rs3751812, rs1558902, rs17817449 | obesity, childhood obesity, young-onset extreme overweight, overweight | Dina Nat Genet 2007, Hinney PLOS One 2007, Meyre Nat Genet 2009, Costapas HMG 2009, Scherag PLOS Genet 2010, Paternoster PLOS One 2011, Wang PLOS One 2011, Bradfield Nat Genet 2012, Berndt Nat Genet 2013, Wheeler Nat Genet 2013 |
| MC4R | rs17782313, rs17700144, rs663129, rs571312, rs476828 | extreme obesity, obesity, overweight | Meyre Nat Genet 2009, Scherag PLOS Genet 2010, Bradfield Nat Genet 2012, Berndt Nat Genet 2013, Wheeler Nat Genet 2013 |
| PCSK1 | rs6232 | obesity | Benzinou Nat Genet 208 |
| PCSK1 | rs6234/rs6235 | obesity | Benzinou Nat Genet 208 |
| FAIM2 | rs7132908, rs7138803 | young-onset extreme overweight, obesity, overweight | Paternoster PLOS One 2011, Bradfield Nat Genet 2012, Berndt Nat Genet 2013 |
| MAF | rs1424233 | extreme obesity | Meyre Nat Genet 2009 |
| NPC1 | rs1805081 | extreme obesity | Meyre Nat Genet 2009 |
| SDCCAG8 | rs12145833 | childhood obesity | Scheragh PLOS Genet 2010 |
| TNKS | rs17150703 | childhood obesity | Scheragh PLOS Genet 2010 |
| KCNMA1 | rs2116830 | obesity | Jiao BMC Med Genomics 2011 |
| BDNF | rs988712, rs10767664 | obesity, overweight | Jiao BMC Med Genomics 2011 |
| TMEM18 | rs4854344, rs2867125, rs6548238, rs12463617 | childhood obesity, obesity, overweight | Bradfield Nat Genet 2012, Berndt Nat Genet 2012, Berndt Nat Genet 2013, Wheeler Nat Genet 2013 |
| POMC | rs6752378, rs10182181, rs713586 | childhood obesity, obesity, overweight | Bradfield Nat Genet 2012, Berndt Nat Genet 2013 |
| TNNI3K | rs1040070 | childhood obesity | Bradfield Nat Genet 2012 |
| SEC16B | rs10913469, rs543874 | childhood obesity, obesity, overweight | Bradfield Nat Genet 2012, Berndt Nat Genet 2013 |

| | | | |
|---|---|---|---|
| OLFM4 | rs9568856, rs9568867 | childhood obesity, obesity | Bradfield Nat Genet 2012, Berndt Nat Genet 2013 |
| HOXB5 | rs9299 | childhood obesity | Bradfield Nat Genet 2012 |
| GPR120 | rs116454156 | obesity | Ichimura Nature 2012 |
| HS6ST3 | rs7989336 | obesity | Berndt Nat Genet 2013 |
| ZZZ3 | rs17381664 | obesity | Berndt Nat Genet 2013 |
| GNAT2 | rs17024258 | obesity | Berndt Nat Genet 2013 |
| HNF4G | rs4735692 | obesity, overweight | Berndt Nat Genet 2013 |
| MRPS33P4 | rs13041126 | obesity | Berndt Nat Genet 2013 |
| ADCY9 | rs2531995 | obesity | Berndt Nat Genet 2013 |
| RPTOR | rs7503807 | overweight | Berndt Nat Genet 2013 |
| NEGR1 | rs2815752, rs1993709 | obesity, overweight | Berndt Nat Genet 2013, Wheeler Nat Genet 2013 |
| GNPDA2 | rs10938397 | obesity, overweight | Berndt Nat Genet 2013 |
| TFAP2B | rs987237, rs734597 | obesity, overweight | Paternoster PLOS One 2011, Berndt Nat Genet 2013 |
| TMEM160 | rs3810291 | obesity | Berndt Nat Genet 2013 |
| ETV5 | rs9816226 | obesity, overweight | Berndt Nat Genet 2013 |
| QPCTL | rs2287019 | obesity, overweight | Berndt Nat Genet 2013 |
| MTCH2 | rs3817334 | obesity, overweight | Berndt Nat Genet 2013 |
| SH2B1 | rs7359397 | obesity, overweight | Berndt Nat Genet 2013 |
| GPRC5B | rs12444979 | obesity, overweight | Berndt Nat Genet 2013 |
| MAP2K5 | rs2241423 | obesity, overweight | Berndt Nat Genet 2013 |
| LRRN6C | rs10968576 | obesity | Berndt Nat Genet 2013 |
| TNNI3K | rs1514174, rs1514175 | obesity | Berndt Nat Genet 2013 |
| RPL27A | rs11042023 | obesity | Berndt Nat Genet 2013 |
| FLJ35779 | rs2112347 | obesity, overweight | Berndt Nat Genet 2013 |
| FANCL | rs887912 | obesity, overweight | Berndt Nat Genet 2013 |
| CADM2 | rs13078807 | overweight | Berndt Nat Genet 2013 |
| NRXN3 | rs10150332 | obesity | Berndt Nat Genet 2013 |
| PRKCH | rs1957894 | childhood obesity | Wheeler Nat Genet 2013 |
| LEPR | rs11208659 | childhood obesity | Wheeler Nat Genet 2013 |
| PACS1 | rs564343 | childhood obesity | Wheeler Nat Genet 2013 |
| RMST | rs11109072 | childhood obesity | Wheeler Nat Genet 2013 |
| NEGR1 | rs3101336 | childhood obesity | Wheeler Nat Genet 2013 |
| LPIN2 | rs643507 | adult obesity in asthmatic subjects | Melen Clin Exp Allergy 2013 |

## Supplementary Table S7. Selected SNPs associated with BMI

| | | RS1 | | | | RS2 | | | | SHIP | | | |
|---|---|---|---|---|---|---|---|---|---|---|---|---|---|
| **Gene** | **SNP ID** | **Coded** | **Ref** | **Freq** | **Rsq** | **Coded** | **Ref** | **Freq** | **Rsq** | **Coded** | **Ref** | **Freq** | **Rsq** |
| FTO | rs9930506 | A | G | 0,58 | 0,99 | A | G | 0,57 | 0,99 | G | A | 0,45 | 1,00 |
| MC4R (1) | rs17782313 | T | C | 0,75 | 1,00 | T | C | 0,74 | 1,00 | C | T | 0,25 | 1,00 |
| MC4R (2) | rs7227255 | G | A | 0,97 | 0,96 | G | A | 0,98 | 0,96 | A | G | 0,02 | 0,94 |
| TMEM18 | rs6548238 | C | T | 0,83 | 1,00 | C | T | 0,83 | 1,00 | C | T | 0,82 | 1,00 |
| GNPDA2 | rs10938397 | A | G | 0,59 | 1,00 | A | G | 0,58 | 1,00 | G | A | 0,45 | 0,99 |
| SH2B1 | rs7498665 | A | G | 0,59 | 0,99 | A | G | 0,58 | 0,99 | G | A | 0,42 | 0,98 |
| KCTD15 | rs11084753 | G | A | 0,67 | 0,94 | G | A | 0,68 | 0,95 | G | A | 0,68 | 1,00 |
| MTCH2 | rs10838738 | A | G | 0,66 | 1,00 | A | G | 0,67 | 1,00 | G | A | 0,35 | 1,00 |
| NEGR1 | rs2815752 | A | G | 0,59 | 0,97 | A | G | 0,57 | 0,97 | A | G | 0,63 | 1,00 |
| SEC16B | rs10913469 | T | C | 0,80 | 1,00 | T | C | 0,82 | 1,00 | C | T | 0,18 | 0,98 |
| ETV5 | rs7647305 | C | T | 0,79 | 1,00 | C | T | 0,80 | 1,00 | C | T | 0,79 | 0,98 |
| BDNF | rs6265 | C | T | 0,81 | 1,00 | C | T | 0,80 | 1,00 | T | C | 0,20 | 1,00 |
| FAIM2 | rs7138803 | G | A | 0,63 | 1,00 | G | A | 0,63 | 1,00 | A | G | 0,42 | 1,00 |
| TFAP2B | rs987237 | A | G | 0,83 | 1,00 | A | G | 0,83 | 1,00 | G | A | 0,20 | 1,00 |
| NRXN3 | rs10150332 | T | C | 0,79 | 1,00 | T | C | 0,78 | 1,00 | C | T | 0,22 | 1,00 |
| GPRC5BB | rs12444979 | C | T | 0,85 | 0,99 | C | T | 0,85 | 1,00 | T | C | 0,15 | 1,00 |
| POMC | rs713586 | C | T | 0,47 | 1,00 | C | T | 0,48 | 1,00 | C | T | 0,47 | 1,00 |
| MAP2K5 | rs2241423 | G | A | 0,77 | 1,00 | G | A | 0,76 | 1,00 | A | G | 0,22 | 1,00 |
| GIPR | rs11671664 | G | A | 0,90 | 1,00 | G | A | 0,90 | 1,00 | A | G | 0,11 | 0,81 |
| FANCL | rs887912 | C | T | 0,70 | 1,00 | C | T | 0,71 | 1,00 | C | T | 0,73 | 1,00 |
| TNNI3K | rs1514175 | A | G | 0,43 | 1,00 | A | G | 0,44 | 1,00 | G | A | 0,59 | 1,00 |
| LRRN6C | rs10968576 | A | G | 0,69 | 1,00 | A | G | 0,71 | 1,00 | G | A | 0,31 | 1,00 |
| FLJ35779 | rs2112347 | T | G | 0,63 | 0,98 | T | G | 0,64 | 0,99 | G | T | 0,37 | 0,99 |
| SLC39A8 | rs13107325 | C | T | 0,95 | 0,94 | C | T | 0,95 | 0,93 | T | C | 0,08 | 1,00 |
| TMEM160 | rs3810291 | G | A | 0,32 | 0,80 | G | A | 0,33 | 0,82 | A | G | 0,67 | 0,90 |
| CADM2 | rs13078807 | A | G | 0,80 | 1,00 | A | G | 0,79 | 1,00 | G | A | 0,20 | 0,99 |
| LRP1B | rs2890652 | T | C | 0,82 | 0,98 | T | C | 0,82 | 0,98 | C | T | 0,19 | 1,00 |
| PRKD1 | rs11847697 | C | T | 0,96 | 0,97 | C | T | 0,95 | 0,97 | T | C | 0,04 | 0,96 |
| MTIF3 | rs4771122 | A | G | 0,74 | 0,91 | A | G | 0,74 | 0,91 | A | G | 0,72 | 0,96 |
| PTBP2 | rs1555543 | C | A | 0,58 | 0,98 | C | A | 0,56 | 0,98 | C | A | 0,57 | 1,00 |
| TUB | rs4929949 | T | C | 0,48 | 0,99 | T | C | 0,49 | 0,99 | C | T | 0,49 | 1,00 |
| HMGA1 | rs206936 | A | G | 0,79 | 0,98 | A | G | 0,80 | 0,99 | G | A | 0,19 | 1,00 |
| CDKAL1 | rs2206734 | C | T | 0,83 | 1,00 | C | T | 0,83 | 1,00 | T | C | 0,18 | 1,00 |
| PCSK1 | rs261967 | A | C | 0,60 | 1,00 | A | C | 0,59 | 1,00 | C | A | 0,44 | 1,00 |
| GP2 | rs12597579 | C | T | 0,95 | 1,00 | C | T | 0,96 | 1,00 | T | C | 0,04 | 1,00 |
| KLF9 | rs11142387 | A | C | 0,47 | 0,99 | A | C | 0,46 | 0,99 | C | A | 0,54 | 0,98 |
| TOMM40 | rs2075650 | A | G | 0,85 | 1,00 | A | G | 0,86 | 1,00 | G | A | 0,16 | 0,69 |

| | | | | | | | | | | | | | |
|---|---|---|---|---|---|---|---|---|---|---|---|---|---|
| FANCL/FLJ 30838 | rs12617233 | C | T | 0,61 | 0,97 | C | T | 0,60 | 0,96 | C | T | 0,59 | 1,00 |
| NTRK2 | rs1211166 | A | G | 0,79 | 1,00 | A | G | 0,79 | 1,00 | A | G | 0,79 | 1,00 |
| GALNT10 | rs7708584 | A | G | 0,43 | 1,00 | A | G | 0,42 | 1,00 | G | A | 0,58 | 1,00 |
| MIR148A/NFE2L3 | rs10261878 | C | A | 0,94 | 0,98 | C | A | 0,94 | 0,99 | C | A | 0,95 | 0,98 |
| ADCY3 | rs7586879 | C | T | 0,66 | 1,00 | C | T | 0,66 | 1,00 | T | C | 0,33 | 1,00 |
| BRE | rs116612809 | A | G | 1,00 | 0,82 | A | G | 1,00 | 0,91 | G | A | 0,00 | 0,94 |
| MAP2K3 | rs11652094 | C | G | 0,71 | 0,98 | C | G | 0,71 | 0,95 | C | G | 0,71 | 1,00 |

Coded, coded allele

Ref, Reference allele

Freq, frequency of coded allele

Rsq, Imputation quality

## Supplementary Table S8. Selected SNPs associated with obesity

| | | RS1 | | | | RS2 | | | | SHIP | | | |
|---|---|---|---|---|---|---|---|---|---|---|---|---|---|
| **Gene** | **SNP ID** | **Coded** | **Ref** | **Freq** | **Rsq** | **Coded** | **Ref** | **Freq** | **Rsq** | **Coded** | **Ref** | **Freq** | **Rsq** |
| FTO | rs1421085 | T | C | 0,62 | 1,00 | T | C | 0,60 | 1,00 | C | T | 0,43 | 1,00 |
| MC4R | rs17782313 | T | C | 0,75 | 1,00 | T | C | 0,74 | 1,00 | C | T | 0,25 | 1,00 |
| PCSK1 (1) | rs6232 | T | C | 0,93 | 1,00 | T | C | 0,94 | 1,00 | C | T | 0,05 | 0,83 |
| PCSK1 (2) | rs6234 | G | C | 0,73 | 0,98 | G | C | 0,73 | 0,99 | C | G | 0,26 | 1,00 |
| FAIM2 | rs7132908 | G | A | 0,62 | 0,98 | G | A | 0,62 | 0,96 | A | G | 0,43 | 0,98 |
| MAF | rs1424233 | T | C | 0,50 | 1,00 | T | C | 0,50 | 1,00 | C | T | 0,51 | 1,00 |
| NPC1 | rs1805081 | T | C | 0,59 | 1,00 | T | C | 0,60 | 1,00 | C | T | 0,43 | 0,99 |
| SDCCAG8 | rs12145833 | T | G | 0,83 | 1,00 | T | G | 0,83 | 0,99 | G | T | 0,16 | 1,00 |
| TNKS | rs17150703 | G | A | 0,90 | 1,00 | G | A | 0,90 | 1,00 | A | G | 0,10 | 1,00 |
| KCNMA1 | rs2116830 | G | T | 0,83 | 0,92 | G | T | 0,83 | 0,95 | T | G | 0,18 | 1,00 |
| BDNF | rs988712 | G | T | 0,77 | 0,98 | G | T | 0,75 | 0,98 | T | G | 0,25 | 1,00 |
| TMEM18 | rs4854344 | T | G | 0,83 | 1,00 | T | G | 0,83 | 1,00 | T | G | 0,82 | 1,00 |
| POMC | rs6752378 | A | C | 0,46 | 1,00 | A | C | 0,47 | 1,00 | A | C | 0,46 | 0,99 |
| TNNI3K | rs1040070 | G | C | 0,44 | 0,99 | G | C | 0,44 | 0,99 | C | G | 0,58 | 0,95 |
| SEC16B | rs10913469 | T | C | 0,80 | 1,00 | T | C | 0,82 | 1,00 | C | T | 0,18 | 0,98 |
| OLFM4 | rs9568856 | G | A | 0,86 | 0,98 | G | A | 0,86 | 0,97 | A | G | 0,12 | 1,00 |
| HOXB5 | rs9299 | T | C | 0,64 | 1,00 | T | C | 0,63 | 1,00 | T | C | 0,66 | 0,98 |
| GPR120 | rs116454156 | G | A | 0,99 | 0,37 | G | A | 0,99 | 0,42 | A | G | 0,02 | 0,70 |
| HS6ST3 | rs7989336 | A | G | 0,48 | 0,99 | A | G | 0,49 | 0,99 | A | G | 0,47 | 1,00 |
| ZZZ3 | rs17381664 | T | C | 0,59 | 0,97 | T | C | 0,59 | 0,96 | C | T | 0,40 | 0,96 |
| GNAT2 | rs17024258 | C | T | 0,97 | 0,99 | C | T | 0,97 | 0,99 | T | C | 0,04 | 0,98 |
| HNF4G | rs4735692 | G | A | 0,41 | 0,98 | G | A | 0,42 | 0,97 | G | A | 0,41 | 1,00 |
| MRPS33P4 | rs13041126 | T | C | 0,73 | 1,00 | T | C | 0,74 | 0,99 | C | T | 0,25 | 1,00 |
| ADCY9 | rs2531995 | C | T | 0,36 | 1,00 | C | T | 0,39 | 1,00 | T | C | 0,62 | 1,00 |
| RPTOR | rs7503807 | A | C | 0,55 | 0,99 | A | C | 0,55 | 1,00 | C | A | 0,45 | 1,00 |
| NEGR1 | rs2815752 | A | G | 0,59 | 0,97 | A | G | 0,57 | 0,97 | A | G | 0,63 | 1,00 |
| GNPDA2 | rs10938397 | A | G | 0,59 | 1,00 | A | G | 0,58 | 1,00 | G | A | 0,45 | 0,99 |
| TFAP2B | rs987237 | A | G | 0,83 | 1,00 | A | G | 0,83 | 1,00 | G | A | 0,20 | 1,00 |
| TMEM160 | rs3810291 | G | A | 0,32 | 0,80 | G | A | 0,33 | 0,82 | A | G | 0,67 | 0,90 |
| ETV5 | rs9816226 | T | A | 0,83 | 1,00 | T | A | 0,83 | 0,99 | T | A | 0,82 | 0,98 |
| QPCTL | rs2287019 | C | T | 0,80 | 1,00 | C | T | 0,81 | 0,94 | T | C | 0,21 | 0,74 |
| MTCH2 | rs3817334 | C | T | 0,61 | 0,99 | C | T | 0,62 | 0,99 | T | C | 0,40 | 1,00 |
| SH2B1 | rs7359397 | C | T | 0,60 | 0,95 | C | T | 0,59 | 0,96 | T | C | 0,42 | 0,98 |
| GPRC5B | rs12444979 | C | T | 0,85 | 0,99 | C | T | 0,85 | 1,00 | T | C | 0,15 | 1,00 |
| MAP2K5 | rs2241423 | G | A | 0,77 | 1,00 | G | A | 0,76 | 1,00 | A | G | 0,22 | 1,00 |
| LRRN6C | rs10968576 | A | G | 0,69 | 1,00 | A | G | 0,71 | 1,00 | G | A | 0,31 | 1,00 |
| TNNI3K | rs1514174 | C | T | 0,44 | 1,00 | C | T | 0,45 | 1,00 | T | C | 0,58 | 1,00 |
| RPL27A | rs11042023 | C | T | 0,66 | 1,00 | C | T | 0,65 | 1,00 | C | T | 0,65 | 0,99 |

| | | | | | | | | | | | | | |
|---|---|---|---|---|---|---|---|---|---|---|---|---|---|
| FLJ35779 | rs2112347 | T | G | 0,63 | 0,98 | T | G | 0,64 | 0,99 | G | T | 0,37 | 0,99 |
| FANCL | rs887912 | C | T | 0,70 | 1,00 | C | T | 0,71 | 1,00 | C | T | 0,73 | 1,00 |
| CADM2 | rs13078807 | A | G | 0,80 | 1,00 | A | G | 0,79 | 1,00 | G | A | 0,20 | 0,99 |
| NRXN3 | rs10150332 | T | C | 0,79 | 1,00 | T | C | 0,78 | 1,00 | C | T | 0,22 | 1,00 |
| PRKCH | rs1957894 | G | T | 0,90 | 0,99 | G | T | 0,90 | 0,99 | G | T | 0,91 | 0,95 |
| LEPR | rs11208659 | T | C | 0,92 | 1,00 | T | C | 0,91 | 1,00 | C | T | 0,10 | 0,99 |
| PACS1 | rs564343 | A | G | 0,41 | 0,99 | A | G | 0,41 | 0,98 | G | A | 0,55 | 1,00 |
| RMST | rs11109072 | C | A | 0,96 | 0,89 | C | A | 0,95 | 0,55 | A | C | 0,04 | 0,99 |
| LPIN2 | rs643507 | T | C | 0,98 | 0,92 | T | C | 0,98 | 0,94 | T | C | 0,98 | 1,00 |

Coded, coded allele

Ref, Reference allele

Freq, frequency of coded allele

Rsq, R-square

**Supplementary Table S9 Selected SNPs associated with *H. pylori*[1]**

| | | | | RS1 and RSII | | SHIP | |
|---|---|---|---|---|---|---|---|
| **Gene** | **SNP ID** | **Minor allele** | **MAF** | **OR (95% CI)** | **p-value** | **OR (95% CI)** | **p-value** |
| TLR10 | rs10004195 | A | 0.25 | 0.74 (0.67-0.81) | 7.42E-10 | 0.62 (0.54-0.72 | 4.95E-11 |
| FCGR2A | rs368433 | C | 0.16 | 0.76 (0.66-0.87) | 1.33E-04 | 0.67 (0.55-0.81) | 2.40E-05 |


1 Mayerle, J. *et al.* Identification of genetic loci associated with Helicobacter pylori serologic status. *JAMA* **309**, 1912-1920 (2013).
